# Yoga in the emergency room: termination of SVT with the Child's pose

**DOI:** 10.1093/omcr/omaf049

**Published:** 2025-05-28

**Authors:** Mohamed Zuhair, Samuel Seitler, Nandita Kaza, Phang Boon Lim, Daniel Keene, Raj Khiani

**Affiliations:** Imperial College London, National Heart and Lung Institute, Hammersmith Hospital, DuCane Road, London, United Kingdom; Royal Free London NHS Foundation Trust, Pond Street, London, United Kingdom; Royal Free London NHS Foundation Trust, Pond Street, London, United Kingdom; Imperial College London, National Heart and Lung Institute, Hammersmith Hospital, DuCane Road, London, United Kingdom; Imperial College London, National Heart and Lung Institute, Hammersmith Hospital, DuCane Road, London, United Kingdom; Imperial College London, National Heart and Lung Institute, Hammersmith Hospital, DuCane Road, London, United Kingdom; Royal Free London NHS Foundation Trust, Pond Street, London, United Kingdom; Royal Free London NHS Foundation Trust, Pond Street, London, United Kingdom

**Keywords:** supraventricular tachycardia (SVT), vagal manoeuvres, yoga therapy, atrioventricular nodal Re-entrant tachycardia (AVNRT), non-invasive cardiac management, autonomic nervous system modulation, Valsalva

## Abstract

We present the case of a 27-year-old woman who has successfully self-managed her supraventricular tachycardia (SVT) episodes since her teenage years using the Child's Pose, a technique adapted from yoga. This non-pharmacological approach enabled her to independently terminate SVT episodes, a capability confirmed in a controlled clinical setting where the patient's return to sinus rhythm was immediately achieved upon assuming the pose. The Child's Pose represents a solitary and patient-empowering intervention, contrasting with the assistance-dependent modified Valsalva manoeuvre. This case demonstrates the potential for yoga-influenced practices in the personalized management of cardiac arrhythmias.

## Introduction

Supraventricular tachycardia (SVT) is a common cardiac arrhythmia that can significantly affect patients' quality of life. Pharmacological management with AV nodal blocking agents such as adenosine is the prevailing strategy for the diagnosis and termination of supraventricular arrhythmias. However, these require administration in a hospital setting and are associated with a significant, albeit short-lived, side effect profile. Nonetheless, there is an increasing interest in non-pharmacological interventions that utilize physiological processes to terminate the arrhythmia. This case report describes a patient with a history of SVT who has self-managed her condition using a yoga posture without medical intervention.

## Case history/examination

The subject of this case is a 27-year-old female midwife with a medical history of recurrent SVT episodes. Her first presentation of SVT was at the age of 12, but it was not until the age of 15 that she discovered the use of a yoga manoeuvre called the Child's Pose, allowed her to self-terminate her SVT. She observed that by adopting the Child's Pose, she could consistently terminate the SVT episodes. This self-initiated approach has been her primary mode of managing these episodes, which she reports occur roughly once a month.

**Figure 1:**
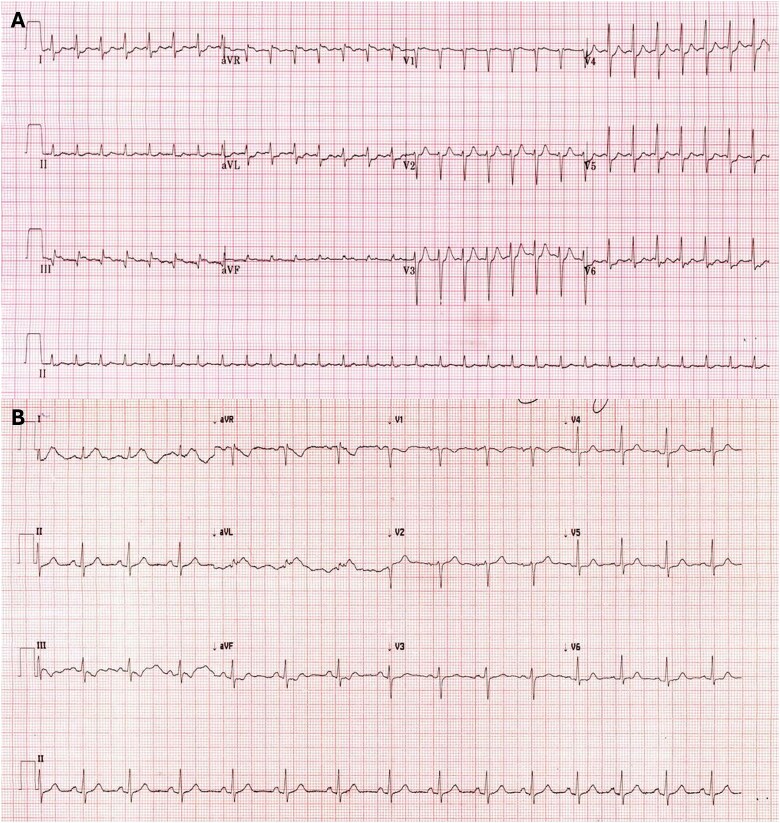
(a) ECG on admission demonstrating AVNRT (b) ECG following patient assuming Child’s pose demonstrating sinus tachycardia.

On presentation to the emergency department, the patient was found to be in an SVT episode with a maximum recorded heart rate of 200 beats per minute, most likely consistent with AVNRT from the ECG showing an RSR' pattern in V1 (Fig. 1a). Under cardiac monitoring, she demonstrated her Child's Pose yoga position, which terminated the SVT within 30 seconds of her assuming the pose. The electrocardiogram captured before and after the intervention revealed an immediate return to sinus tachycardia upon assuming the Child's Pose (Fig. 1a and b).

## Methods (differential diagnosis, investigations and treatment)

To further aid in the dissemination of this technique, the patient consented to a video recording of her performing the Child's Pose to terminate an SVT episode. This video (Video 1) is accessible for educational purposes, illustrating the real-time effectiveness of the manoeuvre. She remained under observation for one hour following restoration of sinus rhythm, before being discharged home. Routine laboratory tests were performed on admission to hospital, and were all within normal limits, including markers for infection (white cell count, C-reactive protein), renal function and electrolytes.

## Conclusion and results (outcome and follow-up)

The patient has been evaluated for a more permanent solution to her condition and was offered an ablation. However, given her ability to terminate her symptoms with this technique, she did not opt for any intervention. She continues to manage her symptoms with this yoga position and does not need to use her PRN flecainide, which was also prescribed on discharge.

## Discussion

**Figure 2:**
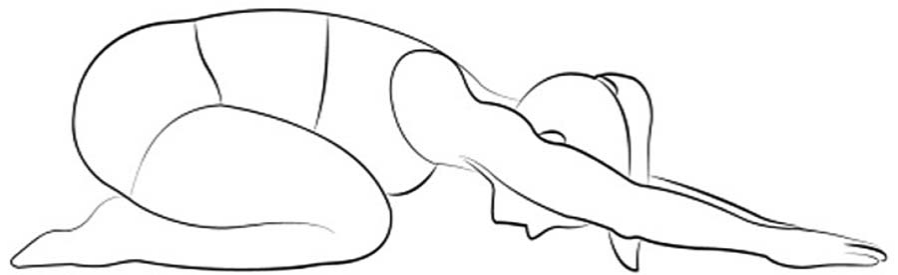
Child’s pose, also known as Balasana.

The Child's Pose is characterized by a forward bend on a stable surface with arms fully extended in front, forehead resting on the surface between the upper arms. The spine is rounded while resting on fully flexed knees in a kneeling position (Fig. 2). In this position, yoga practitioners can feel a deep sense of "resting," particularly when also focusing on breathing deeply, rhythmically, and slowly.

What we propose is that once the child’s pose is assumed, the patient slows and deepens breathing before taking a deep and long inhalation, holding before exhaling through the mouth, ideally with a sigh. This offers the highest chance of vagal activation sufficient to terminate SVT. Furthermore, the forward flexion during this manoeuvre may increase venous return, an additional proposed mechanism for SVT termination. This mechanism may be similar to a modified Valsalva manoeuvre. However, unlike the modified Valsalva manoeuvre, which requires another person to elevate the patient's legs, the Child's Pose is a self-administered technique that can be performed without assistance, providing patients with a sense of control and independence during episodes. Although our patient did not exhibit significant anxiety, it is well known that anxiety can worsen arrhythmias by increasing sympathetic drive, which yoga’s relaxation techniques may also help mitigate.

Studies of yoga's role in arrhythmia management have suggested that yoga practices can reduce heart rate, blood pressure, and psychological stressors, which are significant contributors to arrhythmic events [1–3]. The YOGA My Heart Study demonstrated that in patients with paroxysmal atrial fibrillation, twice weekly yoga can reduce AF burden, symptomatic episodes, and improve patient quality of life^3^. The authors propose yoga to increase baseline parasympathetic tone through downregulation of the hypothalamic–pituitary–adrenal axis, thereby reducing AF symptoms and burden. A similar case of a child with Wolff-Parkinson-White syndrome reverting SVT by performing a handstand corroborates the potential of yoga-related manoeuvres in arrhythmia management [4].

This case report is the first to document clear cessation of SVT with patient-initiated postural adaptation in the form of the Child's Pose. Our proposed mechanism of action suggests that the Child's Pose stimulates the vagus nerve, leading to a deceleration of atrioventricular (AV) node conduction, thereby potentially interrupting atrioventricular nodal re-entrant tachycardia (AVNRT). This proposed mechanism shares similarities with other modified vagal manoeuvres, such as carotid sinus massage and the modified Valsalva manoeuvre.

## Conclusion

This case highlights the innovative application of the Child's Pose, a yoga technique, for the acute termination of supraventricular tachycardia (SVT) episodes. Supraventricular tachycardias (SVTs) are prevalent arrhythmias among younger patients. Incorporating the use of yoga manoeuvres presents a promising addition to home-based management strategies for SVT patients whilst they await more definitive treatment. This expansion of available techniques provides patients with a broader toolkit for self-care and may contribute to improved symptom management and quality of life. Further research is required into the mechanisms and feasibility of other patient-initiated postural techniques, such as Child's Pose, in the initial management of supraventricular arrhythmias.

### Key Clinical message

This case highlights the potential of the Child's Pose, as an effective non-pharmacological approach to managing supraventricular tachycardia (SVT). By promoting vagal stimulation, it provides a patient-centred, self-administered alternative to traditional interventions, giving patients greater autonomy and control over symptom management.

## Supplementary Material

Yoga_in_the_Emergency_Room_omaf049
